# Prevalence and incidence of celiac disease in patients with rheumatoid arthritis: a case-control study based on the RECORD cohort

**DOI:** 10.3389/fmed.2024.1470855

**Published:** 2025-01-20

**Authors:** Garifallia Sakellariou, Annalisa Schiepatti, Anna Zanetti, Carlomaurizio Montecucco, Federico Biagi, Carlo Alberto Scirè

**Affiliations:** ^1^Department of Internal Medicine and Therapeutics, Università di Pavia, Pavia, Italy; ^2^Istituti Clinici Scientifici Maugeri IRCCS, Pavia, Italy; ^3^Gastroenterology Unit, Istituti Clinici Scientifici Maugeri IRCCS, Pavia, Italy; ^4^Epidemiology Research Unit, Italian Society for Rheumatology, Milan, Italy; ^5^Division of Rheumatology, Fondazione IRCCS Policlinico San Matteo, Pavia, Italy; ^6^School of Medicine, University of Milano Bicocca, Milan, Italy

**Keywords:** rheumatoid arthritis, coeliac disease, epidemiology, comorbidity, disability

## Abstract

**Background:**

The reported prevalence of coeliac disease (CD) in rheumatoid arthritis (RA) is variable.

**Objective:**

To evaluate the prevalence and incidence of CD in RA and controls.

**Design:**

Case-control study on administrative data.

**Methods:**

The RECord linkage On Rheumatic Disease database (administrative data, 2004–2013) was used to retrieve patients with RA and age and sex-matched controls. Prevalence and incidence of CD were calculated and stratified according to age, gender, and calendar year.

**Results:**

The cohort included 346,956 subjects (mean age 59.9 (14.5), 70.7% females), of which 70,061 RA and 276,895 controls. Median follow-up was 9 years (IQR 9–9). The prevalence of CD was higher in RA (171/70,061 = 0.24% (0.2–0.3%) vs 398/276895 = 0.14% (0.1–0.2%), *p* < 0.001). The prevalence of CD among females with RA was increased compared to controls (0.3% vs 0.08%, *p* < 0.001), but was not increased in males with RA. The incidence was higher in RA and remained stable throughout the observation period.

**Conclusion:**

The prevalence and incidence of CD were increased in RA, particularly in females.

## 1 Introduction

Coeliac disease (CD) is a chronic immune-mediated enteropathy, triggered by dietary gluten in genetically susceptible individuals and characterized by heterogeneous features (1, 2). The prevalence of CD is around 1% in the general population, and has been increasing over the last decades (3). The clinical manifestations of CD include not only severe malabsorption, but also milder extra-intestinal signs and symptoms, or even pauci-symptomatic/asymptomatic subjects (1, 3). Moreover, patients affected by other disorders such as type 1 diabetes, autoimmune thyroid disorders, chronic iron-deficiency anemia, unexplained infertility and recurrent miscarriages, have an increased prevalence of CD compared to the general population Therefore, in these high-risk groups, testing for CD is recommended, regardless of whether gastrointestinal symptoms are present or not (1, 3–6).

Rheumatoid arthritis (RA) is a systemic inflammatory immune-mediated disease, affecting primarily the synovial joints but extra-articular manifestations are also possible. Patients with RA present an high burden of comorbidities, as well as an increased prevalence of concurrent autoimmune diseases (7, 8).

The association of CD with rheumatological disorders is controversial, as some studies have suggested an increased seroprevalence of CD in rheumatological patients, including patients with RA, while others have found contrasting results (9–19). Recently, a large UK population-based study on the incidence of multiple autoimmune diseases reported an association between RA and CD, diagnosed on the basis of the international classification of the diseases (ICD) codes (8). Although this study found an association between RA and CD, there was limited information on patient characteristics, and no indication on which subgroups of patients were at higher risk. For these reasons rheumatological diseases are not currently included among the high-risk groups in need of serological screening for CD (1–3).

Therefore, the primary aim of our study was to evaluate the prevalence and incidence of CD in a large retrospective cohort composed of patients affected by RA, compared with age-matched controls. For this purpose, we used the RECord linkage On Rheumatic Disease (RECORD) database, derived from administrative claims records, which has been previously used for other studies (20, 21).

## 2 Materials and methods

### 2.1 Study design, population and setting

This is a retrospective cohort study, based on administrative data, aiming to evaluate the prevalence and incidence of CD in adults with RA compared with matched controls. The study cohort was based on the RECord linkage On Rheumatic Disease (RECORD) database of patients with RA, promoted by the Italian Society of Rheumatology (SIR). This is a retrospective cohort based on the Administrative healthcare database (AHD) of Lombardy Region in Italy, which has a population of more than 10 million inhabitants. The RECORD cohort includes patients with RA, included in the AHD between January 1st 2004 and December 31st 2013. The study was promoted by the SIR.

### 2.2 Data collection from AHD

Data on the population of interest was extracted from the AHD, which encompasses people living in the Lombardy region, followed-up until death or transfer to another Italian region or abroad. The database reports demographic information (date of birth, gender, date of death or transfer), drug prescriptions, certifications for chronic and rare diseases, outpatient and inpatient hospital discharge forms, with codification in accordance with the International Classification of Disease, 9th revision, Clinical Modification (ICD-9-CM) diagnoses and Disease Related Group included in the inpatient discharge forms. This database has already been used for previous studies on patients with RA (20, 21). Due to the administrative nature of the data, detailed clinical information on individual patients, such as body weight, dietary habits or serological status, disease activity, which might be relevant to CD and RA, was not available. For this study, the databases reporting demographics and certifications for chronic and rare diseases were used.

### 2.3 Case identification

Patients with RA were identified through an algorithm, including the rheumatologist certification of disease (co-payment exemption code 006.714.0), in keeping with previous studies that demonstrated a high specificity (96.39%) and sensitivity (77.08%) for detecting the disease (12). For each RA patient, 4 age and sex-matched controls from the general population were selected. The observation of patients with RA started at the date of first registration of the exemption code and ended at the date of death, of transfer or December 31st 2013. The observation period for the controls started on January 1st 2004 and ended at the date of death, transfer or December 31st 2013. Patients in the control group that were diagnosed with RA during follow-up were subsequently included among the cases.

### 2.4 Study outcome

The study outcome was defined as a diagnosis of CD. Patients affected by CD were identified through the co-payment exemption code for rare disease RI0060, issued by gastroenterologists, adopted by the ADH throughout the all the study period.

### 2.5 Statistical analysis

Descriptive analyses were performed in patients with RA and controls, reporting mean and standard deviation (sd), median and interquartile range (IQR) and proportions, as appropriate. To characterize our population, we also described the prevalence of other autoimmune diseases with an available exemption code, in particular autoimmune thyroiditis (exemption code 056), Basedow’s disease (035), inflammatory bowel diseases (009) and myasthenia gravis (034). The prevalence of CD in patients with RA and controls was assessed and compared in the two groups by Chi squared, with a level of significance of 0.05. The analysis was repeated after stratifying for age at enrollment and gender. Odds ratios (OR), along with 95% confidence intervals (CI) for the risk of CD in patients with RA compared to controls were calculated for the overall population and stratified by gender. Moreover, we evaluated the incidence of CD in patients with RA and controls, accounting only for new diagnoses of CD separately for each calendar year, to evaluate whether this figure changed over time. All the analyses were performed with R Statistical Software (Foundation for Statistical Computing, Vienna, Austria).

### 2.6 Ethics

Access to the data for the purpose of this study was granted by the General Directorate of Health, in accordance with national ethical requirements. The study complies with the Declaration of Helsinki. The protocol was approved by the ethics committee of the Pavia University Hospital, no specific consent to participate was required.

## 3 Results

### 3.1 Descriptive analysis

The study included overall 346,956 subjects, with a mean (sd) age of 59.9 (14.5) years, of which 245,344 (70.7%) were females and 101,622 males (29.3%). The median (IQR) follow-up was 9 (9–9) years.

Among these, the RECORD cohort included 70,061 RA patients, with a mean (sd) age at the beginning of the observation period of 60 (14.6) years and female predominance (F 49,464, 70.6%). The cohort included 28,089 RA patients at the beginning of the observation period and 58,596 patients at the end. There were 41,972 incident cases of RA and a total of 11,465 deaths in this group (16.36%).

The control cohort consisted of 280,244 subjects with a mean (sd) age of 59.9 (14.5) years, of which 195,880 (70.7%) were female. The control population included 276,895 subjects at the beginning of the observation period and 237,568 at the end, with 39,327 deaths (14.20%).

The prevalence of autoimmune comorbidities at the beginning of observation is reported in Table 1. Patients with RA presented more frequently with autoimmune thyroiditis and myasthenia gravis compared to controls, while the prevalence of the remaining comorbidities was comparable.

**TABLE 1:** Prevalence of other autoimmune diseases in patients with RA and controls.

	RA (*n* = 70061)	Controls (*n* = 276895)
Autoimmune thyroiditis (*n*, %)	1391 (2)	3435 (1.24)
Basedow’s disease (*n*, %)	596 (0.85)	1940 (0.70)
Inflammatory bowel diseases (*n*, %)	265 (0.37)	770 (0.28)
Myasthenia gravis (*n*, %)	74 (0.10)	115 (0.04)

### 3.2 Prevalence and incidence of CD

Within the observation period, a diagnosis of CD based on the exemption code was reported in 171 (0.24%) patients with RA, and in 398 (0.14%) among controls (*p* < 0.001). The OR (95% CI) of receiving a CD diagnosis in patients with RA compared to controls was 1.70 (1.43, 2.04).

The prevalence of CD was higher in females, both among RA patients and controls (RA 0.1% in males vs 0.3% in females, *p* < 0.0001; controls 0.08% in males vs 0.17% in females, *p* < 0.0001).

**FIGURE 1:**
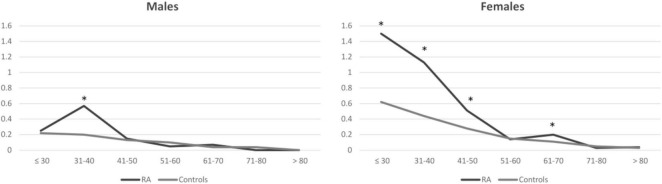
Prevalence of celiac disease in patients with rheumatoid arthritis and controls. Results are presented as percentages and stratified for gender and age classes at the beginning of the observation period. Asterisks correspond to significant results (*p* < 0.05). RA, rheumatoid arthritis.

When comparing patients with RA and controls, the proportion of females with CD was significantly different, being over-represented in RA [0.3% in RA vs 0.17% in controls, *p* < 0.001; OR (95% CI) 2.15 (0.133, 3.48)], while this difference was not seen in male subjects (0.1% in RA vs 0.08% in controls, p 0.292, OR (95% CI) 1.23 [0.76, 1.99]). Moreover, while the prevalence of CD was substantially similar in RA and controls in males throughout the age range (except for patients aged 31–40, in which it was higher in patients with RA), in female subjects, especially at younger ages, there was a significant difference between patients with RA and controls throughout the age range (Figure 1 and Table 2).

**TABLE 2:** Prevalence of CD in patients with RA and controls, stratified according to sex and age.

	Males			Females		
**Age range**	**RA (*n*, %)** **(*n* = 20595)**	**Controls (*n*, %)** **(*n* = 81015)**	***P***	**RA (*n*, %)** **(*n* = 49464)**	**Controls (*n*, %)** **(*n* = 195880)**	***p***
≤30	2 (0.25)	7 (0.2)	1	27 (1.50)	45 (0.62)	<0.001
31–40	10 (0.57)	14 (0.20)	0.017	43 (1.13)	67 (0.44)	<0.001
41–50	4 (0.15)	14 (0.13)	0.771	33 (0.51)	73 (0.28)	0.006
51–60	2 (0.05)	17 (0.1)	0.400	14 (0.14)	61 (0.15)	0.875
61–70	4 (0.07)	9 (0.04)	0.314	28 (0.20)	60 (0.11)	0.009
71–80	0 (0)	6 (0.04)	0.357	3 (0.03)	20 (0.05)	0.603
>80	0 (0)	0 (0)	–	1 (0.04)	3 (0.03)	1

**FIGURE 2:**
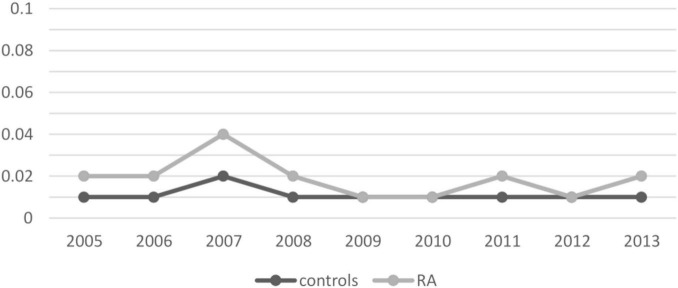
Incidence of celiac disease per calendar year. Results are presented as percentages.

We then assessed the incidence of CD in patients with RA and controls for each calendar year of observation. Overall, the incidence of CD did not increase significantly between 2005 and 2013, ranging from 0.02 to 0.04 in RA patients and from 0.01 to 0.02 in controls, although a two-fold higher prevalence was found in 2007. In general, the incidence of CD was higher in patients with RA compared with unaffected controls throughout the observation period (Figure 2 and Table 3).

**TABLE 3:** Incidence of CD in patients with RA and controls, reported per calendar year.

Year	2005	2006	2007	2008	2009	2010	2011	2012	2013
RA	5/28068 (0.02)	7/32843 (0.02)	16/37138 (0.04)	7/41425 (0.02)	6/45450 (0.01)	7/49218 (0.01)	10/52850 (0.02)	6/56360 (0.01)	10/59562 (0.02)
Controls	38/318684 (0.01)	37/309347 (0.01)	71/300387 (0.02)	42/290971 (0.01)	27/281412 (0.01)	28/271882 (0.01)	26/262257 (0.01)	37/252437 (0.01)	26/242646 (0.01)

## 4 Discussion

This large cohort study based on administrative data of the Lombardy region, Italy, evaluated the prevalence and incidence of CD in a population of adult patients with RA compared with age and sex matched controls, followed-up for a long period of time.

We found that the prevalence of CD in RA is two-fold-higher compared to controls, in line with a recent report, in which an incident rate ratio of between 1 and 1.9 was found (8). The risk in our cohort was consistent with this finding but slightly higher, possibly due to a different age composition of our control group, whose demographic features were matched to those of RA, with lower risk of development of CD, thus increasing the existing gap, also considering a possible under-reporting of CD in the elderly (22). Although patients with RA might have a greater likelihood of being tested for CD, the consistency of our results with previous findings suggests only a limited impact of a potential detection bias.

In addition, we found that this difference in prevalence was mostly related to a higher occurrence of CD in females with RA, while the prevalence in men was not consistently increased. As the prevalence of autoimmune diseases is generally higher in females, it seems that sex acts as an additional risk factor even in subjects already affected by an autoimmune pathology. However, during our period of observation, the excess risk related to female sex remained stable, in contrast with studies reporting an increase of CD diagnoses in females over time (8). A higher degree of medical awareness among women may also explain our results.

In our population, we identified a higher prevalence of CD in females with RA compared to female controls, in particular among those below 60 years of age. These features may identify a subgroup of patients at increased risk of developing CD, warranting serological testing and clinical monitoring. Current guidelines support systematic screening for celiac antibodies in high-risk groups, such as in patients affected by type 1 diabetes and autoimmune thyroiditis, while there is less clarity regarding indication for testing patients with rheumatic diseases. Based on our data and previous epidemiological findings, we suggest considering screening for CD in young women with RA (3).

The second major finding is that the incidence of CD remained stable throughout the study period, in both RA patients and controls. This contrasts with recent reports, that suggest the incidence and prevalence of CD have grown in the last decades (3, 7). Reasons for this discrepancy may include the greater median age of our cohort, matched with patients with RA and therefore representing neither the composition of the general population nor that of typical CD cohorts. In line with this consideration, it has been described that the increasing incidence of CD is due to an increase particularly among younger subjects (8). An additional factor leading to increased incidence of autoimmune diseases may be more frequent detection in patients already affected by other autoimmune diseases, possibly due to greater clinician awareness and development of screening programs (8). We found a prevalence of CD of 0.24% in RA patients and 0.14% in controls. While the expected prevalence of CD in the general population has reported to be higher than in our cohort (>1%), this refers to a younger population than that of our cohort (23). The prevalence of CD in the elderly is poorly studied, but our findings are in keeping with the frequency of 0.1% (95% CI 0,0.99) described in previous reports (24).

The overall prevalence of CD in patients over 50 years old is difficult to establish and contrasting data have been reported (3). Therefore, it is difficult to directly compare our results with those of previous studies on the general population, given the significant differences in study design and population.

In our study, both RA and CD were defined by the exemption codes, thus allowing the inclusion of patients with a well-established clinical diagnosis of both diseases. Older studies on concurrent RA and CD were based on seroprevalence of autoantibodies, which does not fully correspond to a definite diagnosis of these conditions. We think that this might be a strength of our study, as it limits the possibility of inaccurate estimates of prevalence (25). This approach had already been adopted in previous studies based on the RECORD cohort, supporting a good quality of the adopted methodology (12, 13).

Our study has some intrinsic limitations, which are due to the study design based on administrative claims. These include limited data on clinical details, which is a typical drawback of administrative data, not allowing for stratification across several relevant clinical variables. Although stratifying only for gender and age represents a limitation, this is a common situation in studies based on AHDs. Moreover, due to the relatively low prevalence of both conditions, the absolute number of patients with RA and concomitant CD was low, which represents a limitation for the assessment of specific outcomes, such as mortality, cancer, cardiovascular events and osteoporotic fractures. This is also relevant for CD, as it has been reported that an older age, such as that seen in our cohort, is a major risk factor for complications (26). Finally, our control population is not fully representative of the general population, as it was matched following the features of RA patients.

In conclusion, we have shown that the prevalence of CD, whose incidence was stable over time in our study, is at least twice as high in patients with RA compared to controls, particularly among females. These results may support serological screening for CD in females affected by RA, although further studies will be necessary to fully clarify this relationship and define the need for systematic screening

## Data Availability

The datasets analyzed for this study can be found, upon reasonable request, from the corresponding author.
